# Reviewing the state of biosensors and lab-on-a- chip technologies: opportunities for extreme environments and space exploration

**DOI:** 10.3389/fmicb.2023.1215529

**Published:** 2023-08-17

**Authors:** Stefano Cinti, Sima Singh, Giovanni Covone, Luca Tonietti, Annarita Ricciardelli, Angelina Cordone, Roberta Iacono, Arianna Mazzoli, Marco Moracci, Alessandra Rotundi, Donato Giovannelli

**Affiliations:** ^1^Department of Pharmacy, University of Naples Federico II, Naples, Italy; ^2^BAT Center-Interuniversity Center for Studies on Bioinspired Agro-Environmental Technology, University of Napoli Federico II, Naples, Italy; ^3^Bioelectronics Task Force at University of Naples Federico II, Naples, Italy; ^4^Department of Physics, University of Naples Federico II, Naples, Italy; ^5^Department of Science and Technology, University of Naples Parthenope, Naples, Italy; ^6^Department of Biology, University of Naples Federico II, Naples, Italy; ^7^Task Force on Microbiome Studies, University of Naples Federico II, Naples, Italy; ^8^NBFC, National Biodiversity Future Center, Palermo, Italy; ^9^Institute of Biosciences and Bioresources, National Research Council of Italy, Naples, Italy; ^10^INAF-IAPS, Istituto di Astrofisica e Planetologie Spaziali, Rome, Italy; ^11^National Research Council–Institute of Marine Biological Resources and Biotechnologies–CNR-IRBIM, Ancona, Italy; ^12^Earth-Life Science Institute, Tokyo Institute of Technology, Tokyo, Japan; ^13^Marine Chemistry and Geochemistry Department, Woods Hole Oceanographic Institution, Woods Hole, MA, United States; ^14^Department of Marine and Coastal Science, Rutgers University, New Brunswick, NJ, United States

**Keywords:** biosensors, point of care, lab on chip, space exploration, extreme environment

## Abstract

The space race is entering a new era of exploration, in which the number of robotic and human missions to various places in our solar system is rapidly increasing. Despite the recent advances in propulsion and life support technologies, there is a growing need to perform analytical measurements and laboratory experiments across diverse domains of science, while keeping low payload requirements. In this context, lab-on-a-chip nanobiosensors appear to be an emerging technology capable of revolutionizing space exploration, given their low footprint, high accuracy, and low payload requirements. To date, only some approaches for monitoring astronaut health in spacecraft environments have been reported. Although non-invasive molecular diagnostics, like lab-on-a-chip technology, are expected to improve the quality of long-term space missions, their application to monitor microbiological and environmental variables is rarely reported, even for analogous extreme environments on Earth. The possibility of evaluating the occurrence of unknown or unexpected species, identifying redox gradients relevant to microbial metabolism, or testing for specific possible biosignatures, will play a key role in the future of space microbiology. In this review, we will examine the current and potential roles of lab-on-a-chip technology in space exploration and in extreme environment investigation, reporting what has been tested so far, and clarifying the direction toward which the newly developed technologies of portable lab-on-a-chip sensors are heading for exploration in extreme environments and in space.

## 1. Introduction

From an anthropocentric perspective, all terrestrial habitats characterized by harsh environmental conditions are identified as extreme environments (Merino et al., 2019). Under these conditions, including extremely high or low pressure, temperature, pH, salinity, and radiation, the lives of humans are severely challenged and their survival is generally restricted to a very limited number of circumstances. Despite this, extreme environments cover a wide portion of Earth’s surface and are also thought to be abundant in our solar system and beyond (Rothschild and Mancinelli, 2001; Ando et al., 2021). Harsh environmental conditions, which are not only related to natural habitats and hostile remote areas, are also often reported in manufacturing, processing and production sectors of diverse industrial branches, such as automotive, aerospace, nuclear, geothermal and oil and natural gas industries (Fahrner et al., 2001; Johnson et al., 2004). In this regard, an industrial harsh environment can affect machinery life cycles, equipment reliability, efficiency, and productivity of the industry itself, and not least the safety of operators (French et al., 2016).

On Earth’s surface, there are numerous natural sites whose environmental conditions are easily assimilable to those of celestial bodies in our solar system (Hestroffer et al., 2019). Sea ice and permafrost in Polar regions, cold seeps, hydrothermal vents, hot springs, fumaroles, mud volcanoes, hyperacidic or saline lakes, deserts, and arid environments, or nuclear contaminated sites, mimicking conditions that could be found on early Earth, strongly resemble the surface of rocky planets, the large icy moons of Saturn and Jupiter, hydrocarbon-rich environments like Titan, etc. [1] (Preston and Dartnell, 2014). *In situ* observations, in addition to the analysis of water, soil, and biofilm samples collected from these environments, can give fundamental clues about the environmental parameter limits, like pH, temperature, pressure, or salinity, in which life can be found on Earth, and thus presumably elsewhere. These environments are perfect scenarios for testing methods, protocols, models, technologies, and hardware useful for studying geosphere-biosphere coevolution (Yadav et al., 2021). In this way, it is possible to detect the biosignatures and biomarkers useful for space exploration. Finding specific types of molecules in these environments could provide clues about the nature of markers to look for in the cosmos. All these aspects make Earth’s extreme environments perfect natural laboratories where to explore the origin (Botta et al., 2017) and the limits of life (Pikuta et al., 2007), as well as excellent testing grounds for technologies with broader applications in various industrial sectors, and that can be used both during the exploration of our planet and beyond.

Space exploration and space biology are entering a new golden age, with the number of ongoing and planned missions, both manned and unmanned, rapidly increasing as reported by NASA.^1^ Our ability to accurately perform *in situ* analytical measurements under harsh environmental conditions on Earth and, in the future, on other planetary bodies will play a key role in enabling deep-space missions (Covone et al., 2021). Current practices for the detection and identification of chemical and biological compounds are essentially based on the use of classical analytical instrumental methods, such as high-performance liquid chromatography (HPLC), inductively coupled plasma atomic emission spectroscopy (ICP-AES), mass spectrometry (MS), atomic absorption spectrometry (AAS), and so on. These techniques represent the gold standard in analytical chemistry, but require instruments of large size and weight, long time, and effort for sample preparation and analysis, and often involve the use of advanced equipment and modern clean rooms. For all these aspects, these techniques are unsuitable for *in situ* real-time analytical detection, especially in remote locations and in space, where reduced footprint and payload are key requirements (Clatterbaugh et al., 2010; Gastoldi and Cinti, 2023).

In extreme environments such as space, human health surveillance is still an unmet goal, and the development of suitable and reliable biosensors is for this purpose a high-priority need. A major breakthrough in the field of analytical chemistry, applied to extreme environments and to space exploration, is the miniaturization and automation of analytical systems that operate on the lab-on-a-chip (LoC) platform. The development of these technologies has been enabled by recent advances in the fields of smart materials, microfluidics, and electronics (Faheem and Cinti, 2023). Furthermore, an increased and more aware use of point-of-care (PoC) diagnostics (Omidfar et al., 2020) and has accelerated the process of digitalizing routine monitoring procedures (Singh et al., 2021). In fact, LoC-based PoC testing systems are portable, efficient and easy-to-use tools for rapid *in situ* detection, diagnosis, and monitoring of disease, which only require droplets of samples, such as blood, urine, or other body fluids such as saliva, sweat, and tears (Singh et al., 2022a). These characteristics make LoC nanobiosensors ideal for applications in all major sectors related to space and remote environments exploration, from astronauts’ health monitoring to environmental analysis for *in situ* resource utilization and biosignature exploration.

The study of extremophiles and extremely hostile locations can answer many questions from the field of evolutionary biology to molecular biology and industrial biology for space exploration. This is in part due to the several significant challenges that can be encountered in studying biological life within extreme environments, such as the need to travel to remote destinations, work in hostile conditions, and face specific dangers associated with studying them. From this perspective, the development of sensors that can be applied to harsh conditions could provide a system for monitoring life in extreme environments and supporting space exploration by humans.

In this Review, we will focus our attention on lab-on-a-chip (LoC) nanobiosensors in the context of extreme environments and space exploration, reporting the state-of-the-art of current applications, and highlighting the possible directions for novel device development aimed at space missions and planetary research.

## 2. Lab-on-a-chip key features

Technologies for exploring space and extreme environments, both in land and underwater, share a number of common features and key requirements, including the *robustness of the detection technology*, *low-cost sensors, small form factor*, and *real-time capabilities*. LoC technology has the advantage of combining all of these properties, representing the top-of-the-line system for the development of innovative sensors suitable for space exploration and extreme environment analysis.

### 2.1. Robustness of detection technologies

Establishing alternative, stable, and selective system design options is a way to reduce the risks of exploring extreme environments. The development of sensors and electronics set up for harsh conditions has recently gained increasing importance in different industrial settings, including automotive, aircraft, process engineering, and deep-sea research (Coyle and Diamond, 2016). Unlike sensors used in most common applications, the manufacture of those suitable for use in harsh environmental circumstances frequently relies on unique technologies, distinct materials, and highly specialized designs, enabling them to withstand the most challenging conditions (Bogue, 2012). However, the sensitivity, stability, and selectivity of detection techniques still need to be improved. Indeed, most of the currently used systems require very long detection times as well as specialized technicians and peculiar processing equipment, which limit their applications. Colorimetric and electrochemical biosensors are currently the best developed and preferred approaches for the detection and monitoring of analytes under extreme environmental conditions, due to their low detection limits, high selectivity and sensitivity, fast response times, and local detection (Xue et al., 2019; Fernandes et al., 2020; Heard and Lennox, 2020).

### 2.2. Production and utilization costs

There is a need for the development of efficient on-demand technologies based on low production and consumption costs. Microfluidic technology has gained particular interest in the field of LoC systems development, mostly due to its low manufacturing costs. Furthermore, microfluidic devices (μFDs) provide a straightforward assay method widely used for efficient analyte detection. Combining μFDs with colorimetric and electrochemical systems will result in a cost-effective solution that could be widely used in the near future (Faheem and Cinti, 2023). For many years, researchers have explored the possibility of using microfluidic systems in planetary science. Some of these experiments have shown that the optimization process can become even more intensive. As part of the ExoMars mission from NASA and the European Space Agency (ESA), Park et al. (2011) developed a miniaturized antibody microarray system using optical fibers and microfluidics to detect life. The size and weight of the fluid management system were reduced by replacing multiple pneumatically operated on–off valves with a single integrated selector valve. Mora et al. (2011) announced the development of a fully integrated microfluidic system that could perform analysis of amino acids by microchip capillary electrophoresis (μCE) with integrated laser-induced fluorescence detection.

### 2.3. Real-time capabilities

Real-time information technology is required in extreme environmental conditions and hazardous circumstances to improve the protection and survival of the involved personnel. Additionally, real-time information is needed to implement autonomous decision-making in the field, enabling the use of artificial intelligence to guide unmanned exploration and support human presence in space. As an example, Hinkov et al. (2022) have recently developed a next-generation model of an optical, fingertip-sized mid-IR LoC device, suitable for sensitive and selective *in situ* real-time analysis of chemical reactions in liquids. In particular, they demonstrated the capability of the device to monitor temperature-induced changes in the secondary structure of proteins in real-time. This technology may find wide use in biomedical applications for the investigation of chemical reactions, such as drug production (Hinkov et al., 2022).

### 2.4. Small form factor

**Figure 1:**
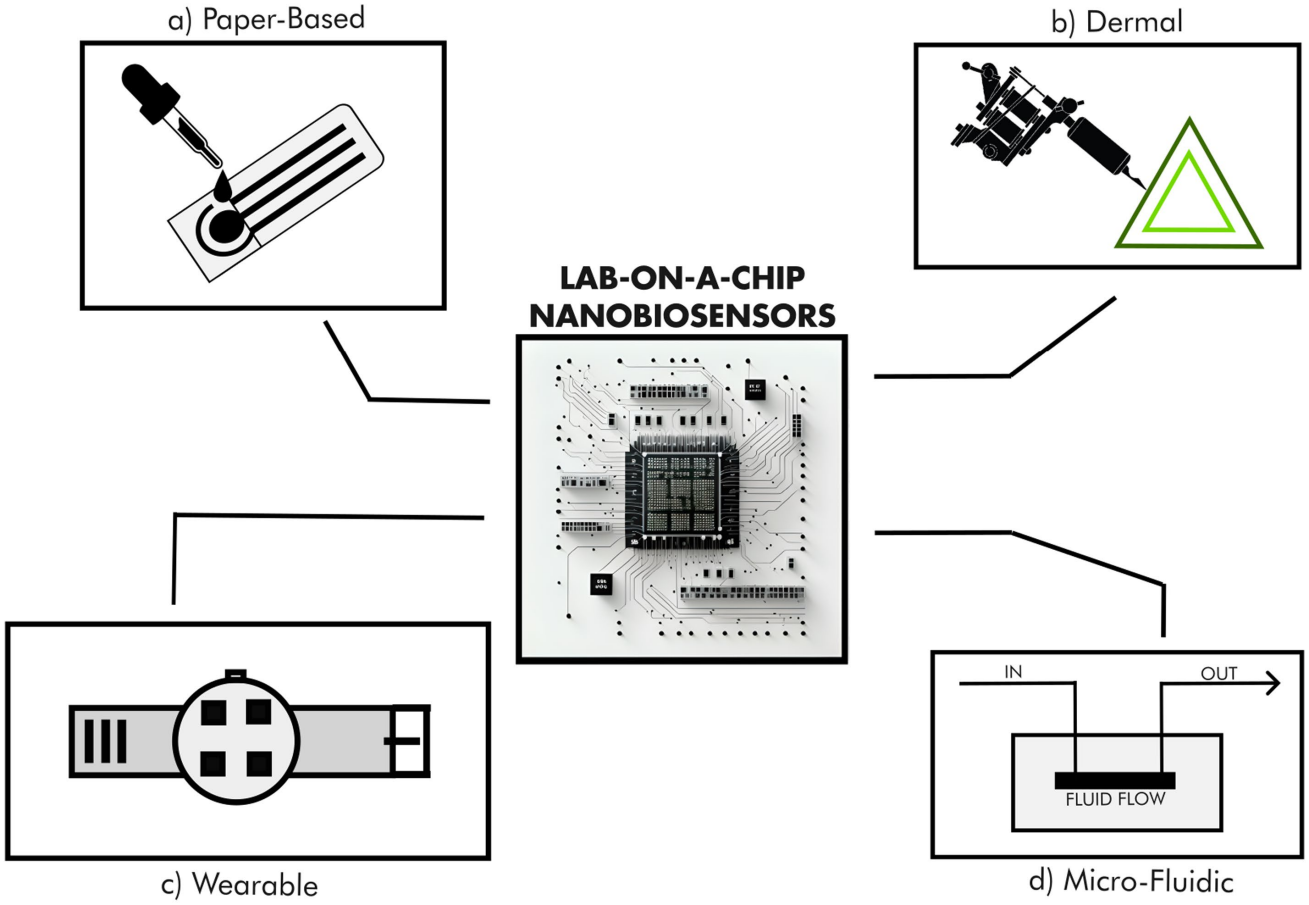
Examples of different lab-on-a-chip nano-biosensors and their technologies: **(A)** Paper-based, **(B)** Dermal, **(C)** Wearable, and **(D)** Micro-fluidic.

**Figure 2:**
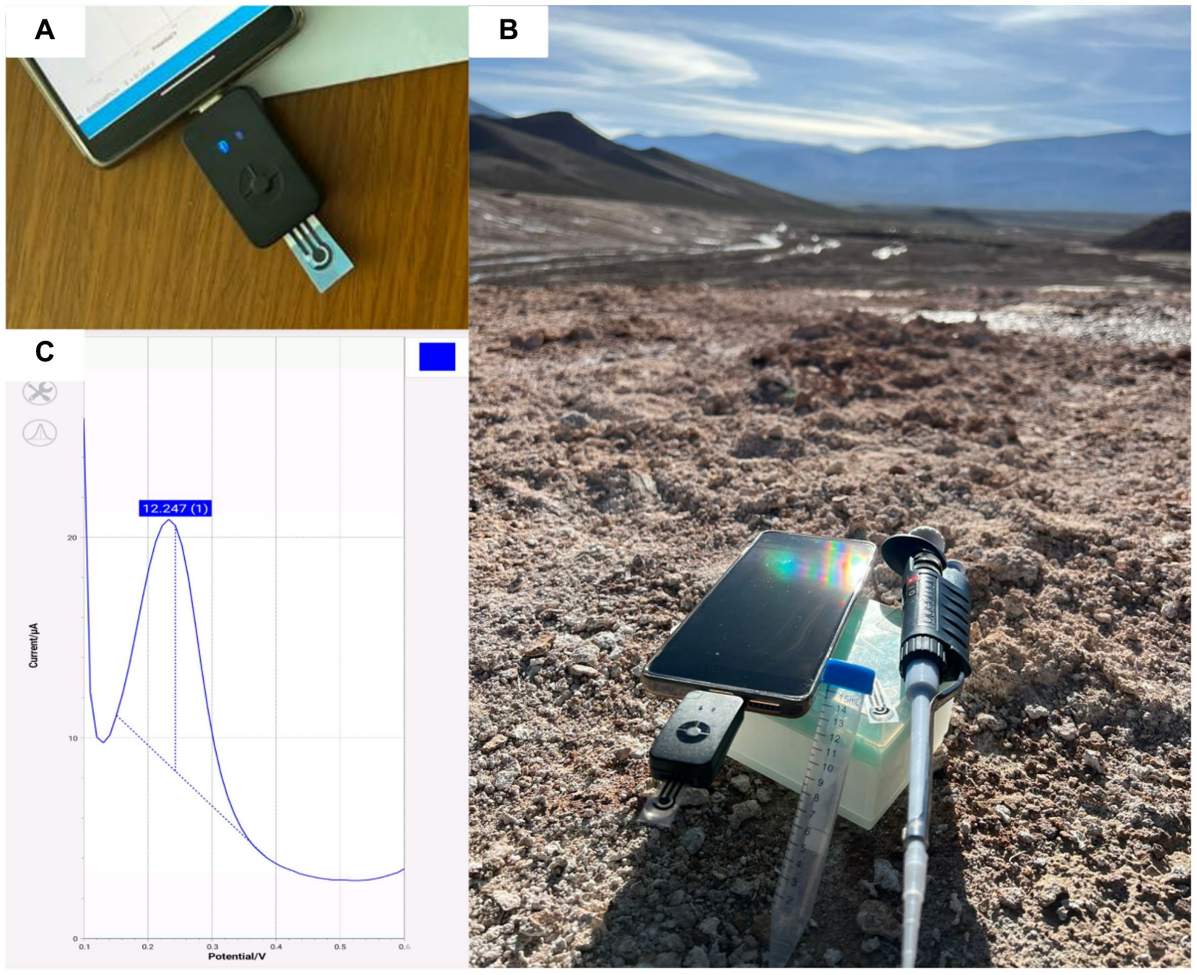
Examples of lab-on-a-chip sensors used in extreme environments. **(A)** Close-up of the paper-based sensor and the reading unit; **(B)** the sensor in the field during recent fieldwork in the Puna plateau (arid desert with altitude above 4,000 meters); and **(C)** example of data collected in the field relatively to the measurement of copper (II) ions directly onto the screen-printed strips using a PalmSens Sensit as a portable readers powered by a smartphone.

In the exploration of remote places on our planet as well as in space, the size and weight of analytical devices are fundamental features for the selection of the instruments to backpack during a mission. Minimizing size and weight not only allows the production of compact detection systems, but also cuts launch costs and power requirements (Hessel et al., 2020). In this sense, LoC devices have the smallest footprint of any analytical alternative (Figure 1). In particular, paper-based microfluidic systems and biosensors have weights several orders of magnitude lower than traditional analytical devices (Figures 1, 2). Moreover, micro-processing provides processing conditions that are easily adaptable to the use of artificial intelligence technologies and, taking place in enclosed chambers with no headspace, allows vacuum operation (Hessel et al., 2021). Ultimately, LoC nanobiosensors represent excellent candidates for space missions, both as probe-installed instruments and as wearable technologies for astronauts. They found wide use in different areas of space biology, from life detection to spacecraft bioburden or astronaut health monitoring. Even though astonishing advances have been made in the LoC sector in the last decade (Cinti, 2019; Cinti et al., 2019; Parolo et al., 2020; Singh et al., 2022b), their application in extreme environments exploration is still poorly investigated.

## 3. Nanobiosensors for extreme environments and space microbiology

The interaction between humans and microbes on Earth is not the same as that in the confined environment of space (Liu et al., 2016). Since astronauts are required to spend extended periods of time in an orbiting spacecraft during a mission in space, the investigation of microbial pathogenesis and infection processes in space could help preserve astronaut health. Based on the results of decadal studies conducted by NASA, a series of microbial tracking experiments have been launched aiming to understand the dynamics of microbial persistence on spacecraft and related microbial processes, including biofilm formation, pathogenicity, and virulence (Bijlani et al., 2021). Conducting experiments in space and transporting samples back to Earth for further studies require a significant investment of time, money, and human effort. Therefore, microbiological risk must be regularly assessed and controlled. These applications require sensors that operate under extreme conditions with long-term accuracy and precision. In this context, nanomaterials are promising candidates for the manufacture of single-molecule nano-biosensors with high-throughput biosensor arrays, with improved electrochemical, mechanical, magnetic, and optical properties (Mehrotra, 2016). The combination of nanostructures with electrochemical processes has led to an increase in the effectiveness, sensitivity, response time, and degradability of the sensors, thus enabling efficient and cost-effective assays for a wide range of analytes. For the same reason, the use of nanobiosensors is rapidly spreading in diagnostics and in the biochip industry (Soy et al., 2022).

In space biology and in astrobiology, one of the main goals is to explore and visit different celestial bodies in our solar system. This approach ultimately serves to determine their potential habitability and the presence/absence of life. Several strategies have been attempted to achieve this: analyzing the biogeochemical properties of meteorites, mimicking planetary atmospheric conditions in lab-scale experiments, or using extreme environments on Earth as natural laboratories and planetary analogs. According to CAREX (Marteinsson et al., 2013; Coordination Action for Research on Study of Life in Extreme Environment), planetary analogs can be described in four main domains: the contribution of life to biogeochemical cycles and the response to environmental variability in extreme environments; the response, adaptation, and evolution of life forms in environments extremely-rich in biotic and abiotic stresses; bioenergetics and biodiversity in extreme environments, and the relationship between life and habitability.^2^ Following these guidelines, the exploitation of extreme environments as planetary analogs could help explain the relationship between life, environments, and humans, in order to better design technical strategies for deep space exploration. An experimental approach of particular interest in space microbiology is rock biomining and/or bioleaching, namely the processes of extracting metals from low-grade ores and wastes through microbial activity (Olsson-Francis and Cockell, 2010; Santomartino et al., 2022). A heterogeneous and complex microflora, composed of both heterotrophic and autotrophic acidophilic microorganisms, is generally used in most industrial biomining processes (Hoque and Philip, 2011). Due to the extreme environmental conditions in which these microorganisms live and perform metal extraction and solubilization, such as high temperatures and very low pH, the development of suitable analytic systems is urgently needed for biomining and bioleaching process monitoring, but more generally for setting up planetary simulation facilities aimed at the study of rocky planets and moons.

Depending on the type of parameter or analyte to be monitored or detected and the environmental conditions under which this needs to occur, analytical systems based on LoC technology have been designed, suitable for the most varied applications (Figure 2). Below are some examples that have been reported, i.e., the electrochemical detection of copper ions at a printed strip.

### 3.1. Sensors for extreme temperature conditions

Sensor performance requirements vary depending on whether they are used in high- or low-temperature conditions. Temperature is probably the environmental parameter that mostly affects the operation of most analytical devices (Bogue, 2012). For example, with standard thermocouples and pyrometers, it is difficult to obtain accurate measurements of the thermal pulse to which nanoparticles and microparticles are exposed under harsh conditions, such as explosions, furnaces, and combustion. Under these conditions, with rapid heating rates and high maximum temperatures, the formation of opaque gasses and liquid streams containing nanoparticles and microparticles limits the precision of these methods (Anderson et al., 2020). To avoid such issues, Kalyakin et al. (2019) have developed an *in situ* approach to monitoring the partial pressure of water vapor in oxidizing atmospheres and at elevated temperatures. They employed a new basic amperometric sensor design for electrolytes based on ZrO_2_ and CaZrO_3_. The electrolyte membranes allow the conductivity of oxygen anions and protons to be established, leading to the complete decomposition of water as a result of the applied potential by an external circuit. The proper performance of the proposed sensor is demonstrated by varying the partial pressure of water vapor (0.003–0.110 atm) in air and temperature (675–750°C). Sensor measurement confirmed test success by proving to be steady, repeatable, and consistent with theoretical predictions and practical applications (Kalyakin et al., 2019).

### 3.2. Sensors for volcanics

The temperature and composition of volcanic gasses fluctuate as a result of geochemical reactions and seismic activity. Volcanic acoustic signals contain important information about shallow magnetic and hydrothermal processes. In certain circumstances, the detection of acoustic waves is the sole unequivocal proof of the presence of volcanic eruptions. Using a microelectromechanical differential pressure transducer, Grangeon and Lesage (2019) detected and analyzed volcanic sonic waves. It has a wide frequency range, a wide dynamic range, low noise level, and comparatively low power. Despite its ease of installation, it is designed for use in extreme environments. The instrumental response of each infrasound sensor is precisely measured from 1 mHz to >100 Hz, providing sinusoidal pressure changes. It is relatively easy to handle and install due to sensor size (26x45x80 mm) and weight 100 g (Grangeon and Lesage, 2019). The discovery of volcanoes may therefore aid in the prediction of phreatic eruptions and other types of eruptions in the near future.

### 3.3. Sensors for environmental microbes

Microorganisms not only thrive in a wide variety of environments on Earth but can also withstand the harsh conditions of space, including high radiation, vacuum pressure, drastic temperature changes, and microgravity (Merino et al., 2019). Real-time monitoring of pollutants, contaminants, and pathogens on multiple geographic scales is essential to adequately assess environmental risks, manage impacts, and understand the processes that determine their extent and spread (Zielinski et al., 2009). Fricke et al. (2019) demonstrated that contemporary isothermal microcalorimeters (IMCs) can detect the metabolic heat of bacteria with enough precision to detect even minute bacterial contamination in water, food, and medical samples. Compared to the culture technique, the IMC technique was reported to be much faster in detecting high bacterial concentrations in turbid solutions without even shaking the sample (Fricke et al., 2019).

### 3.4. Sensor for underwater applications

Due to the extreme environmental conditions found in particular aquatic environments, such as deep oceans, LoC systems used in these areas have special requirements. Compared to land-based equipment, the electrical systems utilized in LoC systems must be able to operate in a water-filled environment and tolerate exceptionally high hydrostatic pressures. LoC analyzers should be used in the presence of severely corrosive salt water and strong hydrostatic pressure. However, the materials, analytical techniques, or operational principles that can be used have some limitations. High levels of automation are also required to enable autonomous operations in remote or extreme environment areas. In recent decades, advances in LoC technology have made underwater analysis and *in situ* detection possible. Colorimetric approaches are often used to assess marine variables such as micro and macronutrient content, such as nitrates, phosphates, and silicates (Worsfold et al., 2013). For example, to perform *in situ* ammonia detection, a device integrating a gas diffusion cell and electrodes was developed for conductivity measurements. The gas diffusion cell is simple in design, with a thin Teflon tape between the polysulfone blocks, which have a serpentine microchannel design (Plant et al., 2009).

### 3.5. Sensors for H^+^ ions for extreme pH measurement

Oceans absorb approximately a quarter of anthropogenic CO_2_ emissions from the atmosphere, thus resulting in a decrease of surface ocean pH, with an average rate of 0.0017 pH units per year, as reported by the Copernicus Marine Environment Monitoring Service (CMEMS).^3^ This ocean acidification phenomenon also affects total dissolved inorganic carbon (DIC) speciation, reducing the amount of carbonate ions in seawater ions available for calcifying organisms to build and maintain their shells, skeletons, and other calcium carbonate structures. To predict changes in marine carbonate chemistry on high spatial and temporal scales from surface waters to the deep ocean, a novel LoC microfluidic sensor for *in situ* pH high-precision measurements was developed. Thanks to LoC technology, this sensor showed 10 times the depth capability of the state-of-the-art autonomous spectrophotometric sensor. Furthermore, the LoC pH sensor demonstrated high-level performance on fixed and moving platforms under varying environmental salinity, temperature, and pressure conditions (Yin et al., 2021).

## 4. Nanobiosensors for astronaut health and life support systems

**Figure 3:**
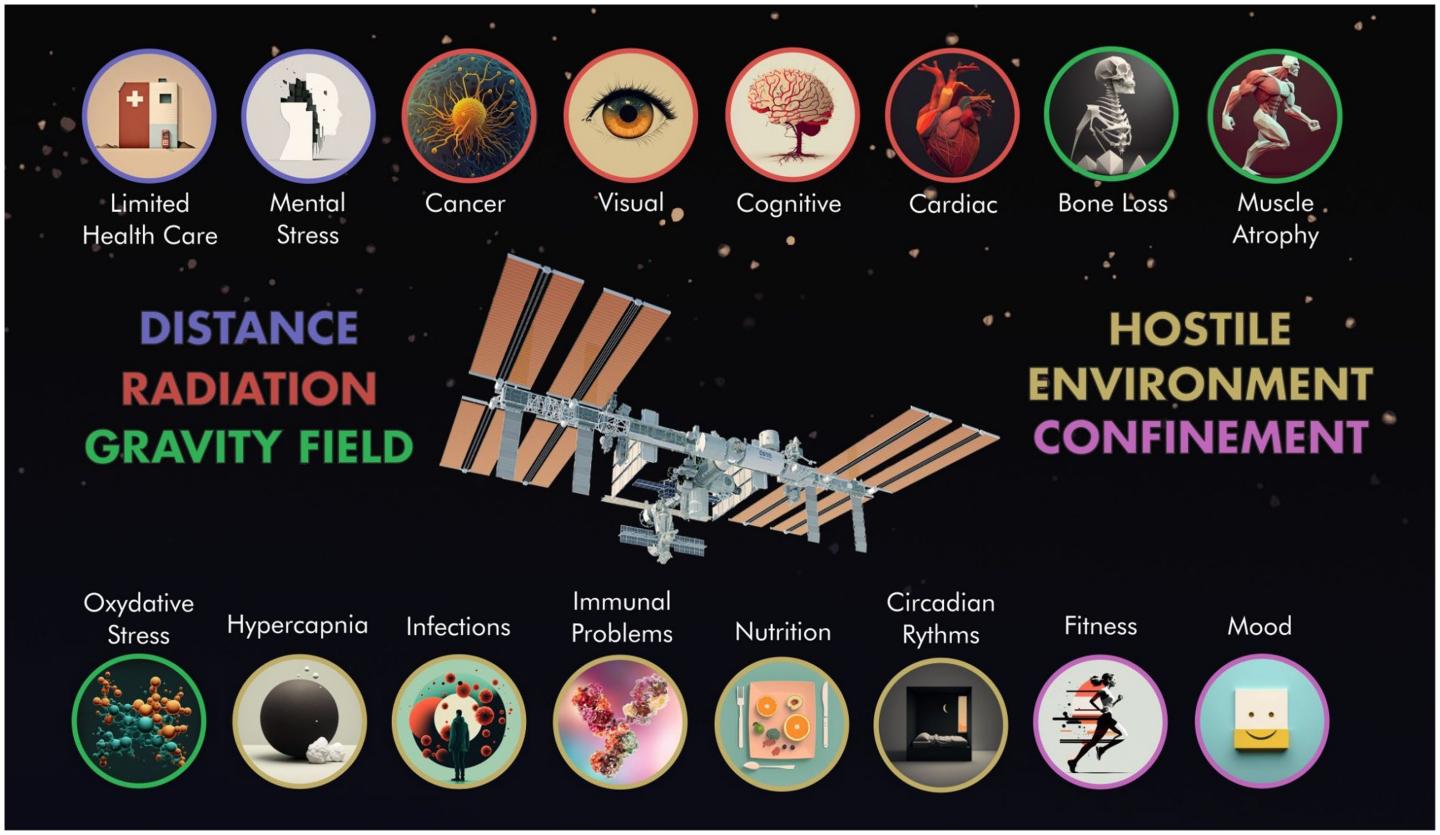
Physical-Biological factors that affect human space exploration and their associated stress: blue circle–distance, red circle–radiation, green circle–gravity field, yellow circle–hostile environment, and pink circle–confinement. Adapted by Barker et al. (2021).

Space is an unnatural and harsh environment that affects the human body in many ways. From the first spaceflight in the early 1960s to current space missions, several microgravity-related alterations in astronauts’ physiology have been reported (Nicogossian et al., 2016). These complex physiological changes, depending on the duration of the spaceflight, range from a change in body fluids, muscle atrophy, bone demineralization, and immune dysregulation (Williams et al., 2009; Liu et al., 2016) to increased radiation exposure (Löbrich and Jeggo, 2019) and potential exposure to pathogens and microbes due to increased bioburden on the spacecraft (Figure 3). Furthermore, the functioning of astronauts’ psychology is also affected (Figure 3; Barker et al., 2021). Taken together, all these factors can negatively influence human health and productivity on the space station (Voorhies et al., 2019). In order to preserve astronauts’ health during missions, it should be possible to perform *in situ* diagnosis, management, and treatment of pathologies related to spaceflight. Today, balanced healthcare systems are based on the 4P medical model, which is preventive, predictive, personalized, and participatory medicine, which forms the basis for advanced patient surveillance technology (Flores et al., 2013). All four requirements of the 4P model for medical and analytical devices that provide clinically relevant information without requiring a core clinical laboratory, are achieved by PoC technology. Recent research has focused on adapting PoC sensing techniques to “resource-constrained environments,” with the automation field considered to be the most important market for PoC electronics in the near future (McCracken and Yoon, 2016; Singh et al., 2021).

Electrochemical and optical technologies combined with miniature readout subsystems are by far the most widely used for PoC sensing. They can be used as wearable, implanted, and *in situ* detection systems. PoC devices can also measure physiological parameters such as heart function rate, oxygen saturation, plasma protein composition level, and patterns of various biomarkers and their concentrations. In addition to reducing detection time, these technologies would also allow testing in locations where laboratory experiments are not feasible, such as in space. In the future, these sensors will be used by humans in space and could pave the way for more sustainable space exploration (Koehne et al., 2020).

### 4.1. Astronaut physiology monitoring during space flight

Monitoring health in space is a tough task to accomplish, due to several issues of different nature. Clear examples are unconventional space environmental conditions, in addition to the lack on the space station of medical devices, such as electrocardiographs, blood pressure machines, fingertip oxygen saturation monitors, and ankle bracelet activity sensors, which are often too heavy, bulky, invasive, disruptive, and expensive. The remarkable advances in biosensors offer the possibility of developing novel diagnostic methodologies based on LoC technology for monitoring the health status of astronauts, which could feasibly allow for the acquisition of personalized therapeutic approaches. As an example, the opportunity to perform *in situ* analysis of blood components or specific biomarkers, such as exosome-like small extracellular vesicles (sEVs) (Tkach and Théry, 2016), and circulating cell-free nucleic acids (*cf*-NAs) (Bezdan et al., 2020), would be useful to identify physiological effects or immune system responses related to microgravity, radiation exposure, or other peculiar spaceflight stress conditions. Several biosensor formats have been proposed as in-flight diagnostic tools, despite their many disadvantages: clinical diagnostic biosensors, biosensors for water and environmental quality, and biosensors for investigating the effects of and countermeasures to radiation exposure (Roda et al., 2018).

Recently, an ultrasensitive electrochemical-digital cortisol sensor chip was developed as a digital stress analyzer for preventive *in situ* monitoring of steroid hormone present in human saliva (Pan et al., 2017). Cortisol is a well-known stress biomarker (Pasha et al., 2014), and its levels in saliva samples were determined using electrochemical impedance spectroscopy (EIS) after the sensor was interfaced and reconfigured using a high-precision impedance converter device. Another example is the use of antibody-based biosensors, the most advanced and most commonly used, such as IMMUNOLAB, a bioanalytical device based on immunological analyzes (Kern and Eisenberg, 2015) that allows chemical clinical analyzes to be performed on blood, urine, or saliva samples onboard the ISS and performs both sample preparations and target detection.

More generally, new-generation biosensor technologies will exploit nanomaterials to improve the mechanical, electrochemical, optical, and magnetic properties of biosensors offering a large range of medical and economic benefits, ranging from disease prevention, improved clinical results, and improved quality of life, reducing the burden of medical care, and providing the necessary analytical performance for space medicine.

### 4.2. Monitoring exposure to radiation

Humans are exposed to radiation from various natural and artificial sources. Ionizing radiation contains enough energy to interact with living cell atoms and cause genetic damage, thus causing long-term human health consequences, such as heart disease and cancer (Stabin, 2022). Spaceflights and long-term space missions expose astronauts to high doses of radiation. Similar conditions can occur on Earth at high altitudes or in specific work environments. To ensure safety under extremely high radiation conditions, a practical and easy-to-access monitoring system is needed. Johary et al. (2021) described the use of sophisticated image sensors in today’s ubiquitous mobile phones that can detect both ionizing radiation and visible light. Smartphone cameras use a sensor called a CMOS (complementary metal oxide semiconductor) to capture images. If the radiation hits the sensor, it generates a signal that can be separated from the visible-light signal and enables the smartphone to be used as a radiation detector. The Roland-Dieter Klein radioactivity counter is available for download in the Apple App Store. The smartphone CMOS sensor is very sensitive to radiation exposure as low as 10 μGy/h (Johary et al., 2021). Similar CMOS sensors might be adapted for in-flight applications that offer relatively simple, inexpensive, and rapid deployment in space. However, it does not guarantee the same performance as its ground-based counterpart. To this end, an example is the TimePIX chip, an innovative and cutting-edge device, emerging as a pivotal instrument in the realm of radiation detection and imaging, particularly in the realm of space exploration. Its remarkable capabilities make it an indispensable tool for the meticulous examination and profound understanding of the intricate radiation environment encountered by intrepid astronauts, ensuring not only their well-being but also the preservation of vital equipment. With its exceptional sensitivity to various radiation particles, the TimePIX chip assumes a pivotal role in meticulous monitoring and meticulous analysis of radiation levels that permeate the vast expanse of space. It provides invaluable insights into discriminating crucial aspects such as particle taxonomy, energy magnitude, and intensity, thereby furnishing scientists with essential parameters and aimed at safeguarding astronauts against formidable potent radiation exposure (Kroupa et al., 2015; Urban et al., 2020, 2023; Urban and Doubravová, 2021).

Alternative radiation detection technologies include a polyacrylamide hydrogel doped with modified boron nitride nanosheets (BNNS-TA) and Fe^3+^ ions (Jiang et al., 2022). The aBNNS-TA/Fe^3+^/PAAm hydrogel exhibits exceptional mechanical properties, pressure sensitivity and γ-ray resistance. The excellent compressive strength, performance recovery, and high sensitivity make it suitable for use as a pressure sensor in a control circuit or as an attachment to the body for accurate detection of human activity, suggesting its use for nuclear safety monitoring, accident management, and extravehicular activities (Jiang et al., 2022).

## 5. Conclusion

One of the main goals of space research is to extend the human experience to the farthest reaches of space to discover, explore, and exploit uncharted regions of space. However, life in space continues to bring new insights into human physiology and psychology that may lead to the failure of current detection systems (Krishen, 2009). Radiation, plasma, atomic oxygen, outgassing, and contamination can drastically limit the life of a space flight and seriously affect the success of a project. Accurate, precise, efficient, and cost-effective health monitoring is consistently important. Biosensor systems are reported to be good analyzers for chemical substance measurements (Haney et al., 2020). However, the combination of many harsh conditions creates significant difficulties for the development of suitable instruments. Lightness, compressibility, self-healing, radiation, micrometeoroids, and orbital debris resistance would be all desirable features in sensors design. In this perspective, LoC-based sensors represent the best choice among the finest detection systems able to detect and respond to harsh situations. They can be optical fibers, nanosensors, or vibration sensors in combination with artificial intelligence and telemetry. Microfluidic technology is a good example and the best way to accelerate processes in science and industry today, and it will soon transform the entire industry (Krishen, 2009; Hessel et al., 2020).

In this review, currently used systems for detection of analytes in extreme environments and space exploration were discussed, suggesting PoC and LoC approaches represent the best choice. They ensure accurate and continuous monitoring of the analytes, without sacrificing selectivity or sensitivity. However, new advances in the fields of engineering, nanomaterials and electronics can improve durability, reversibility, sensitivity, and selectivity of the analytical devices, supporting the development of next-generation systems suitable for harsh environment investigation and space exploration.

## Author contributions

SC and DG conceived the review. All authors contributed to the article and approved the submitted version.

## Funding

This work is partially funded by the European Research Council (ERC) under the European Union’s Horizon 2020 research and innovation program (grant agreement No. 948972) to DG.

## Conflict of interest

The authors declare that the research was conducted in the absence of any commercial or financial relationships that could be construed as a potential conflict of interest.

## Publisher’s note

All claims expressed in this article are solely those of the authors and do not necessarily represent those of their affiliated organizations, or those of the publisher, the editors and the reviewers. Any product that may be evaluated in this article, or claim that may be made by its manufacturer, is not guaranteed or endorsed by the publisher.

## Footnotes

^1^(https://nssdc.gsfc.nasa.gov/planetary/upcoming.html).

^2^ cordis.europa.eu/project/

^3^https://doi.org/10.48670/moi-00224, Accessed on 26 April 2023.
